# Global Prevalence of *Yersinia enterocolitica* in Cases of Gastroenteritis: A Systematic Review and Meta-Analysis

**DOI:** 10.1155/2021/1499869

**Published:** 2021-09-02

**Authors:** Seyed Mohammad Riahi, Ehsan Ahmadi, Tayebeh Zeinali

**Affiliations:** ^1^Cardiovascular Diseases Research Center, Department of Epidemiology and Biostatistics, Birjand University of Medical Sciences, Birjand, Iran; ^2^Student Research Committee, Birjand University of Medical Sciences, Birjand, Iran; ^3^Social Determinants of Health Research Center, Department of Public Health, School of Health, Birjand University of Medical Sciences, Birjand, Iran

## Abstract

The prevalence of *Yersinia enterocolitica* in gastroenteritis is often underestimated. It relates considerably to morbidity and medical expenses around the world. Understanding the cause of gastroenteritis leads to making the appropriate treatment decisions. We systematically searched PubMed, Science Direct, Embase, and Scopus to identify all published studies between Jan. 1, 2000, and Dec. 31, 2019, to assess the prevalence of *Y. enterocolitica* in gastroenteritis patients. A total of 5039 articles were identified that lead to the extraction of data from 47 of them. The pooled prevalence of *Y. enterocolitica* in cases of gastroenteritis was estimated as 1.97% (1.32–2.74%) in the culture method and 2.41% (1.07–4.22%) in the molecular method. Among the biotypes of *Y. enterocolitica*, 1A (62.48%) and 1B (2.14%) had the most and least prevalence, respectively. Serotype O3 *Y. enterocolitica* with 39.46% had the highest and O5,27 with 0.0% had the least prevalence in gastroenteritis cases. In conclusion, the findings of this systematic review show that *Y. enterocolitica* is prevalent in gastroenteritis in all age groups. Serotypes O3 and O9 of *Y. enterocolitica* had the highest prevalence and O5,27 had the least prevalence in diarrheal patients. The prevalence of *Y. enterocolitica* was similar in both gender and different seasons. It should be noted that to determine the role of the organism, more studies are needed especially in food-borne diseases.

## 1. Background

Yersiniosis is caused by Gram-negative bacteria *Yersinia enterocolitica* (*Y. enterocolitica*) and *Y. pseudotuberculosis*. Although *Y. enterocolitica* is a frequent cause of human infection especially in developed countries of temperate zones, *Y. pseudotuberculosis* human infection is rare [1]. It mainly caused a gastrointestinal infection in humans. Additionally, *Y. enterocolitica* can cause other clinical manifestations including mesenteric lymphadenitis, endocarditis, and predominantly infects children [2]. Yersiniosis is the third cause of notifiable bacterial zoonosis in the European Union after campylobacteriosis and salmonellosis [3]. *Y. enterocolitica* is a psychrotrophic organism that can replicate at temperatures ranging from 0 to 44°C. As such, the organism can replicate in the refrigerator and survives in frozen foods and liquids for long periods. Peritrichous flagella causes the motility of *Y. enterocolitica.* Motility is temperature dependent, as the bacterium is motile at 25°C but is not motile when it grows at 37°C. Pathogenesis of *Y. enterocolitica* also depends on temperature. The invasive proteins of *Y. enterocolitica* produce at environmental temperatures of less than 28°C and under acidic conditions at 37°C. The expression of virulence factors necessary to infection initiates by the gradual increase of temperature within the host [2]. Infections caused by *Y. enterocolitica* pathogenic strains do not belong to a specific age group, but the clinical manifestation is frequently observed in children and younger adults. Adults can be asymptomatic carriers of infection [4]. Fever, abdominal pain, and diarrhea are the common symptoms of yersiniosis in children [2]. The bacterium was isolated from domestic and wild animals. Pigs are regarded as the reservoir of the pathogen [5], but high titers of anti-Yersinia antibodies in domestic animals, such as cattle, goats, and sheep revealed that there are other possible sources [6]. The main method of human infection is through consumption of contaminated food especially raw or undercooked ones [2] though drinking of contaminated water, close exposure to pet animals, and blood transfusion have also been mentioned [2, 7]. *Y. enterocolitica* had several biotypes and serotypes. Virulent isolates of *Y. enterocolitica* are attributed to certain biotypes and serotypes. Among the six known biotypes (including 1A, 1B, 2, 3, 4, and 5), 1A is reported as an nonpathogenic biotype in healthy people. *Y. enterocolitica* serotypes O3, O8, O9, and O5. 27 were isolated from most cases of human yersiniosis [2]. The most serious disease is caused by serotype O8 with extensive ulceration of the gastrointestinal tract and sometimes death of the patients [8].

Patients may defecate *Y. enterocolitica* for 90 days after the recovery, which shows the importance of early detection of the bacterium in order to prevent transmission and possible outbreak [9]. In order to detect the *Y. enterocolitica*, a culture method and molecular assays were developed. The conventional culture method is time-consuming and has false-negative results while PCR is not only a sensitive and specific detection method but also is able to identify the pathogenic isolates and further characterization of the isolates [4]. Around the world, there is limited information about the prevalence of yersiniosis due to the clinical presentation of the disease as gastroenteritis so the diagnosis and treatment mainly depend on the clinicians and not on the microbiological culture. The aim of the present study was to estimate the global prevalence of yersiniosis in cases of gastroenteritis. Moreover, the main biotypes and serotypes were determined. The existing data and knowledge were synthesized through a systematic literature review and meta-analysis.

## 2. Methods

### 2.1. Search Strategy and Study Selection

A systematic review was performed in PubMed, Science Direct, Embase, and Scopus to identify all published studies between Jan 1, 2000, and Dec 31, 2019, with the search keywords of “gastroenteritis,” “*Yersinia enterocolitica*,” and “yersiniosis” and related terms without any language restriction. The searched keywords were extracted from the Medical Subject Headings thesaurus. The search strategy was presented in the supplementary file. Titles and abstracts of relevant original articles after the removal of duplicates were screened by two independent reviewers (TZ and EA). The bibliographies of the included articles were hand-searched for additional references. Gray literature was searched by using Google Scholar. PRISMA guidelines were used to perform the systematic reviews.

Selection of studies was carried out by the following criteria: primary research studies including original article either published or in press; studies with a cross-sectional design; case group of case-control studies; studies including detection of *Y. enterocolitica* on the samples based on culture or PCR; patients having the symptoms of gastroenteritis; studies performed in a specified region or country; having a known number of sample size; and studies with available full texts. Studies with confusing text or incomprehensible analyses that did not report the sample size and number or percent of positive cases toward *Y. enterocolitica* were excluded. Reviews, letters, or editorial articles without original data were also excluded.

### 2.2. Data Extraction and Risk of Bias Assessment

A standard dedicated data extraction form was designed in Excel software. Two authors (TZ and SMR) extracted data independently. If provided, the following data were extracted from each study: *bibliographic characteristics*, including first author, year of publication, start and end year of the study, study design (cross-sectional or case-control), and country (income, HDI and WHO region); *population characteristics*, including the age of the participants (mean ± standard deviation (SD), minimum and maximum), gender, and total number of tested patients; *methodological information*, including diagnostic method, number of patients positive for *Y. enterocolitica* in culture and PCR separately, season of sampling, biotypes and some prevalent pathogenic serotypes of isolated *Y. enterocolitica*, and geographic location (latitude and longitude). We included samples with both *Y. enterocolitica* and another pathogen detected (e.g., *E. coli* or viruses).

Data were stratified by the diagnostic method and age. Regarding age, data were stratified into four categories: younger than 6 years, 6 to 18 years old, 18 to 59 years old, and more than 60 years old. As an indicator of development and epidemiological context, income, WHO region, and human development index were used to categorize the data on the basis of the country in which the study was performed. The eligible studies were qualified independently by two authors (TZ and EA) according to the Joanna Briggs Institute [10].

### 2.3. Statistical Analysis

In the current study, random-effect models were used for estimating pooled prevalence and 95% confidence intervals (95% CI). Metaprop command was used in Stata software. Pooled prevalence was calculated using a Freeman–Tukey double arcsine transformation [11, 12]. Heterogeneity among studies was examined by *I*^2^, Cochran's Q. I2 index ranges between 0 and 100 percent and *I*^2^ ˃ 70% was considered heterogeneous [13, 14]. A Forest plot in the random-effects model was applied to show pooled prevalence. Subgroup analysis and metaregression were done to identify the sources of heterogeneity [15]. Univariate metaregression analysis was used for assessing the effect of publication year, human development index, geographical location (longitude/latitude), and quality score on the prevalence of *Y. enterocolitica*. In a subgroup analysis, we estimated the prevalence of *Y. enterocolitica* in different age groups, type of diagnostic method, study's type, income, and WHO regions. Publication bias was not examined because the aim of the study is not to determine the association between exposures and outcome [15]. The significance level was considered 0.05 in all analyses. All analyses were done by using STATA 13 (STATA Corp., College Station, Texas). In the metaregression, *p* value <0.1 was considered as a significant level due to the little range of prevalence of *Y. enterocolitica* and the rare nature of the organism.

## 3. Results

### 3.1. Study Characteristics

**Figure 1:**
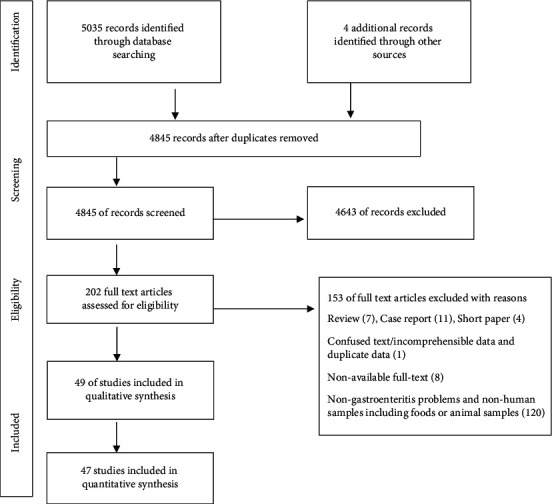
Flowchart of identification and selection of studies for inclusion in the review.

**Figure 2:**
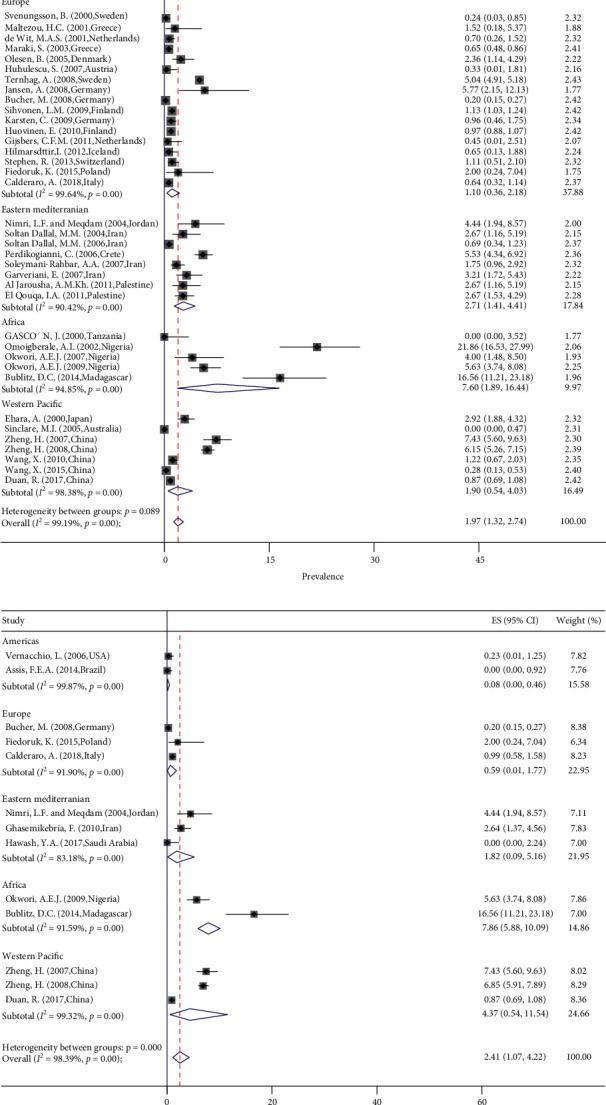
Forest plots for random-effects meta-analysis of the prevalence of *Y. enterocolitica* by (a) culture method and (b) PCR method in WHO regions.

A total of 5039 articles were identified of which 4845 were not duplicates. According to the title and abstract, 202 articles were included and assessed for eligibility by full texts (Figure 1). From these, 49 articles passed the quality assessment and data were extracted from 47 of them. The final extracted data included 25 countries from all WHO regions (eight from the Americas, 17 from Europe, ten from Eastern Mediterranean, five from Africa, and seven from Western Pacific) except for the South-East Asia region. From these 47 studies, the prevalence of *Y. enterocolitica* by culture diagnosis method in cases of gastroenteritis was estimated as 1.97% (95% CI 1.32–2.74; *I*^2^ = 99.19%; *p* < 0.09 test for heterogeneity) (Figure 2(a)). However, by the PCR method the estimate of pooled prevalence for *Y. enterocolitica* was 2.41 (95% CI 1.07–4.22; *I*^2^ = 98.39%; *p* < 0.00 test for heterogeneity) (Figure 2(b)). There was significant heterogeneity among the included studies.  Table 1 shows the pooled prevalence of *Y. enterocolitica* by culture and PCR method according to the countries. The highest prevalence of *Y. enterocolitica* in culture and PCR method was in Madagascar (16.56%). The lowest prevalence of *Y. enterocolitica* in culture and PCR method was in Australia (0.00%) and Brazil (0.00%), respectively.  Table 2 shows the main characteristics of the included studies.

### 3.2. Subgroup Analysis

**Figure 3:**
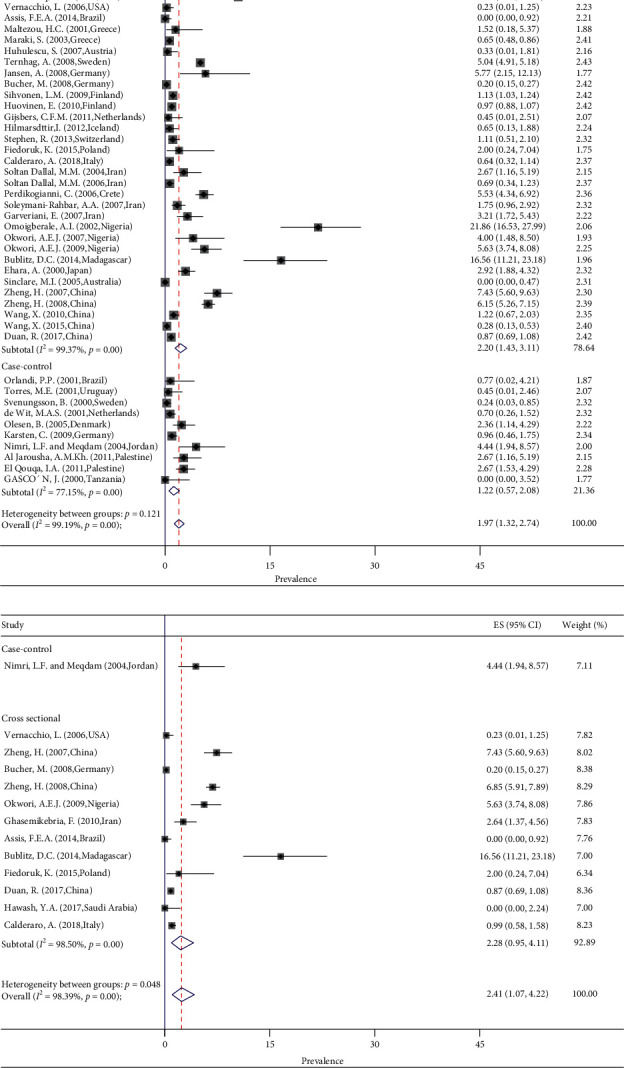
Forest plots for random-effects meta-analysis of the prevalence of *Y. enterocolitica* by (a) culture method and (b) PCR method according to the type of studies.

**Figure 4:**
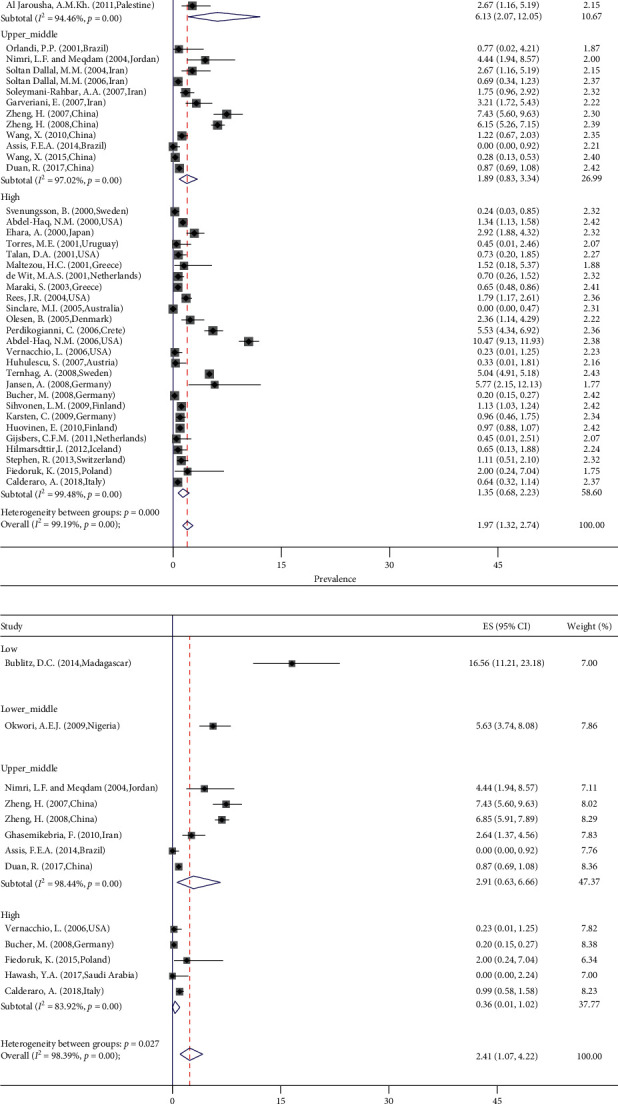
Forest plots for random-effects meta-analysis of the prevalence of *Y. enterocolitica* by (a) culture method and (b) PCR method according to income of countries.

**Figure 5:**
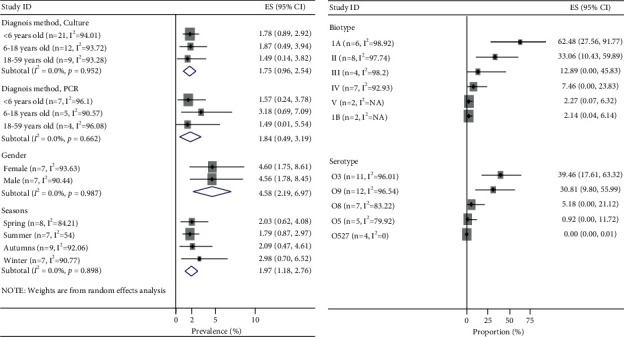
Forest plots for random-effects meta-analysis of the prevalence of *Y. enterocolitica* by (a) age and gender of participants and season of sampling and (b) biotypes and serotypes.

The type of study did not change the pooled prevalence of *Y. enterocolitica*, as in the culture method, the pooled prevalence in the cross-sectional and case-control studies is 2.20 and 1.22, respectively (random test for heterogeneity *p* < 0.12) (Figure 3(a)). The pooled prevalence of *Y. enterocolitica* by the PCR method in the cross-sectional and case-control studies is 2.28 and 4.44, respectively (*p* < 0.05) (Figure 3(b)). The pooled prevalence of *Y. enterocolitica* was decreased by the increase in the income of the countries (*p* < 0.001). The pooled prevalence of *Y. enterocolitica* in low- and high-income countries was 7.17 and 1.35 in culture (*p* < 0.001) (Figure 4(a)) and 16.56 and 0.36 in PCR method (*p* < 0.02) (Figure 4(b)), respectively. The source of heterogeneity of included studies is income. According to age, the prevalence was not significantly different in younger than 6 years, 6–18 years, and 18–59 years (1.75%; 0.96–2.54; *p* < 0.95 for culture and 1.84%; 0.49–3.19; *p* < 0.66 for PCR; *I*^2^ = 0.0%) (Figure 5(a)). By gender of participants and season of sampling, the prevalence was similar (*p* < 0.98 and *p* < 0.89, respectively) (Figure 5(a)). According to the biotype of *Y. enterocolitica* isolates, 1A (62.48%; 95% CI 27.56–91.77) and 1B (2.14%; 95% CI 0.04–6.14) had the most and least prevalence, respectively. Among the investigated serotypes of *Y. enterocolitica* isolates, O3 with 39.46% had the highest and O5,27 with 0.0% had the least prevalence (Figure 5(b)).

### 3.3. Metaregression

**Figure 6:**
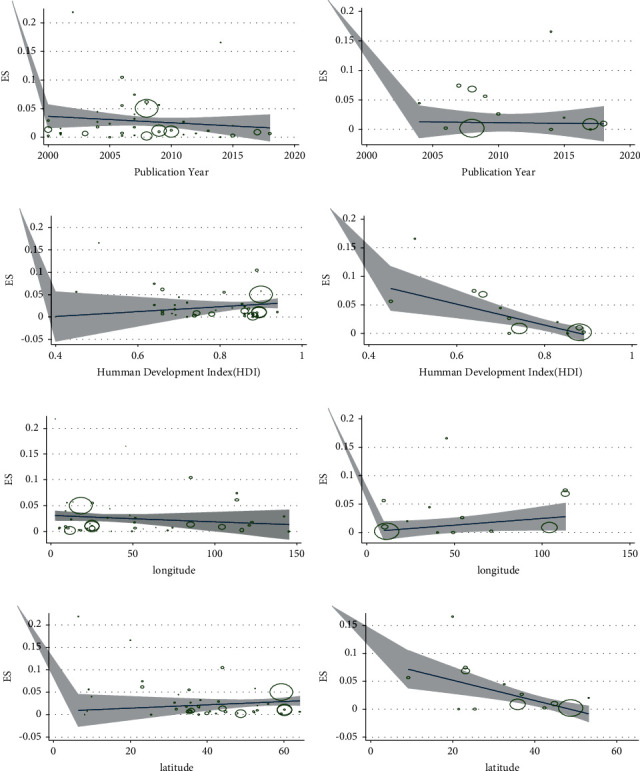
Metaregression results between the prevalence of *Y. enterocolitica* and (a) publication year of the culture studies; (b) publication year of the molecular studies; (c) human development index of the culture studies; (d) human development index of the molecular studies; (e) longitude of the countries of the culture studies; (f) longitude of the countries of molecular studies; (g) latitudes of the countries of the culture studies; (h) latitudes of the countries of molecular studies.

According to Figures 6(a) and 6(b), by the increase of publication year, the prevalence did not have any significant change (*p* < 0.51 for culture and *p* < 0.38 for PCR). Countries with higher HDI had a lower prevalence of *Y. enterocolitica* (*p* < 0.39 for culture and *p* < 0.01 for PCR) (Figures 6(c) and 6(d)). Longitude had not any significant effect on the prevalence of *Y. enterocolitica* (*p* < 0.7 for culture and *p* < 0.24 for PCR) (Figures 6(e) and 6(f)). The prevalence of *Y. enterocolitica* increased slightly with increasing latitude but was not statistically significant (*p* < 0.12) in the culture method; in contrast, its prevalence was decreased with the increasing latitude in the PCR method (*p* < 0.01) (Figures 6(g) and 6(h)). Metaregression for quality assessment and prevalence was carried out and no relation was observed (*p* < 0.74 for culture and *p* < 0.33 for PCR).

## 4. Discussion

In the current meta-analysis, we provided the first estimates of the global prevalence of yersiniosis in cases of gastroenteritis. Based on the culture isolation of *Y. enterocolitica*, Africa [1, 26, 39, 40, 44] and Eastern Mediterranean [17, 22, 42, 46, 48, 53, 56–58] WHO regions had the first and second rank of prevalence of the bacterium, while Europe [4, 16, 19, 21, 24–26, 28, 29, 33–36, 38, 45, 49, 50, 54] had the least prevalence of *Y. enterocolitica* in gastroenteritis cases. Yersiniosis had a global prevalence and is a reportable disease in some countries, such as Denmark, Norway, and 38 states of USA [59, 60]. According to PCR detection, Africa and Western Pacific [3, 9, 18, 23, 27, 37] had the most, and the Americas [20, 30–32, 41, 51, 52, 55] had the least prevalence of *Y. enterocolitica*. In the present study, the highest prevalence of *Y. enterocolitica* in culture and PCR method was in Madagascar (16.56%). The lowest prevalence of *Y. enterocolitica* in culture and PCR method was in Australia (0.00%) and Brazil (0.00%), respectively, in the current study. Bublitz et al. (2014) reported that the prevalence of *Y. enterocolitica* is 16.56% in Madagascar and Assiss et al. (2014) reported it is 0.0% in Brazil. In the United States (US), 0.33 per 100000 individuals were infected by Yersinia during 1996 to 2012 in the general population according to Food-borne Diseases Active Surveillance Network, 2012 [61]. In Denmark, *Y. enterocolitica* was reported as a common cause of bacterial diarrheal disease with 4.9 cases per 100,000 inhabitants in 2016 [62]. Among developed countries, food-borne yersiniosis was higher in most European countries than US [63, 64]. The prevalence of *Y. enterocolitica* was higher in gastroenteritis patients than in the general healthy population. In the present study, income was the origin of heterogeneity among included studies. As, in the low-income countries, *Y. enterocolitica* was more prevalent than high-income ones. This can be related to considering hygiene principles. Human yersiniosis is commonly caused by *Y. enterocolitica* [59]. Yersiniosis caused self-limiting diarrhea that sometimes may be bloody in children younger than four years old. However, fever and abdominal pain accompanied by diarrhea and/or vomiting were reported in older children and adults [9]. The clinical presentation of gastrointestinal disease can be different based on the age and immune status of the host [2]. Diagnosis of yersiniosis is done by isolation of the microbe from human feces or blood or following removal of the appendix, mistakenly [59], although the culture of the bacterium is not a usual procedure for gastrointestinal patients in most hospitals that may lead to underestimates of yersiniosis [59].

Age was not a significant factor regarding gastroenteritis caused by *Y. enterocolitica* in the current study. Some studies reported that younger children are more susceptible to diarrhea caused by *Y. enterocolitica* [9, 27, 39, 40]. Al Jarousha et al. reported higher isolation of *Y. enterocolitica* from diarrheic children with the age of one to six years than children less than one year and more than 6 years [48]. *Y. enterocolitica* had different biotypes and serotypes. The insignificant effect of age may be due to infection of children, adults, and the elderly with different serotypes that may not necessarily create immunity to other serotypes [65]. Furthermore, limited studies were performed on older ages. Gender difference was not seen in the current study. Men and women did not show different symptoms in yersiniosis [40, 49]. A seasonal variation was not seen in the present study. Some studies reported more cases during the cooler season [46, 66], but according to the report of the European Centre for Disease Prevention and Control, no seasonal pattern was observed for yersiniosis for a period of three years [67]. Some other studies did not also report a significant difference between seasons [9, 47, 68], which may support the hypothesis that the infection is transmitted via food items that are consumed consistently throughout the year, such as meat and meat products [65].

Among the six biotypes of *Y. enterocolitica*, 1A was the most prevalent biotype. As biotype 1A is a nonpathogenic biotype mostly found in the environment, it had a higher prevalence in most studies and was isolated from human, animals, and gastroenteritis [49, 50, 69]. Among the virulent biotypes, biotypes II and III had a prevalence of 33.06% and 12.89%, respectively. In the current study, serotypes O3 and O9 had the most prevalence. They were reported in other studies as the main serotypes of *Y. enterocolitica* in diarrheal patients [4, 39, 43]. Serotype O8 was the third serotype in gastroenteritis patients of the current study. It was observed as the most pathogenic serotype in biotype 1B that was correlated to four of six food poisoning outbreaks in the US [41]. A total of 18% of the patients were infected with pathogenic *Y. enterocolitica* [49]. A total of 0.6% of acute diarrhea cases were because of *Y. enterocolitica* and all of them were serotype O3 [54]. In Nigeria, *Y. enterocolitica* bioserotype 2/O9 was the only isolated pathogenic in human samples. Bioserotype 4/O3 of *Y. enterocolitica* is the major isolated one from humans globally [63] and was isolated in some European countries, including Denmark, Italy, Belgium, Spain, Finland, and Sweden [50, 64]. According to Stephen et al., biotypes II and IV were only diagnosed in diarrheal patients, but strains of biotype 1A were isolated from both asymptomatic and diarrheal patients which shows the biotype 1A is not the etiologic agent of gastroenteritis [45]. *Y. enterocolitica* serotype O3 was commonly isolated from children, whereas *Y. enterocolitica* serotype O9 was frequently isolated from adults (≥40 years of age). Exposure of children to *Y. enterocolitica* O3 may conceivably provide some immunity against acute infections due to the same serotype during their life, but not necessarily from other serotypes [65]. According to HDI, the prevalence of *Y. enterocolitica* was increased with the decrease of HDI that can be related to a higher level of hygienic standards in these countries. In the current study, latitude had a different effect on the prevalence of *Y. enterocolitica* in culture and molecular diagnosis. *Y. enterocolitica* is a psychrotrophic bacterium and can replicate in cooler climates [59]. A study on seroprevalence of *Y. enterocolitica* in wild boars showed that the prevalence was higher in cold climates [70]. Similar results were seen in pigs [71]. The viable organisms were detected in the culture method, but in PCR, the not viable ones were also detected which may be the reason for the higher prevalence of *Y. enterocolitica* in the temperate zone in the molecular diagnosis compared to culture. The range of prevalence was narrow in the current study which may be the reason for different observations in culture and PCR method, although, in culture, it was not significant.

### 4.1. Strengths and Limitations

This was the first systematic review and meta-analysis to gain a global prevalence of *Y. enterocolitica* in gastroenteritis patients. We considered both the culture and PCR isolation of the organism. There was high heterogeneity among the studies especially due to income but mostly reduced by the application of subgroup analysis and metaregression. Additionally, this study has some limitations that must be acknowledged: first, in some analyses, the number of included studies was low, especially in the older ages (e.g., >60 years); second, there were not sufficient related studies for assessing risk factors; third, the age of participants was not reported clearly in some included studies. Forth, the transmission method of the organism was not reported in the studies. However, estimating the global prevalence of *Y. enterocolitica* is challenging as most of the studies were performed in hospitalized patients with gastrointestinal symptoms. We encourage further studies, especially in the western Pacific and southeast WHO regions to produce and share local data about yersiniosis. An update of our study should be done due to the availability of additional data.

## 5. Conclusion

In conclusion, the findings of this systematic review show that *Y. enterocolitica* is prevalent in gastroenteritis in all age groups. *Y. enterocolitica* was not prevalent in high-income countries and countries with higher HDI values. Serotypes O3 and O9 of *Y. enterocolitica* had the highest prevalence and O5,27 had the least prevalence in diarrheal patients. The prevalence of *Y. enterocolitica* was similar in both gender and different seasons. It should be noted that to determine the role of the organism, more studies are needed especially in food-borne diseases.

## Figures and Tables

**Table 1:** A pooled prevalence of *Y. enterocolitica* by culture and PCR method according to the countries.

Country	Culture		PCR	
	Number	Pooled prevalence (95% confidence interval)	Number	Pooled prevalence (95% confidence interval)
Global	45	1.97 (1.32–2.74)	13	2.41 (1.07–4.22)
USA	5	2.09 (0.23–5.62)	1	0.23 (0.01–1.25)
Brazil	2	0.03 (0.00–0.53)	1	0.00 (0.00–0.92)
Uruguay	1	0.45 (0.01–2.46)	—	—
Sweden	2	4.95 (4.82–5.08)	—	—
Greece	2	0.50 (0.32–0.69)	—	—
Netherlands	2	0.60 (0.19–1.20)	—	—
Denmark	1	2.36 (1.14–4.29)	—	—
Austria	1	0.33 (0.01–1.81)	—	—
Germany	3	1.09 (0.05–3.11)	1	0.20 (0.15–0.27)
Finland	2	1.05 (0.98–1.12)	—	—
Iceland	1	0.65 (0.13–1.88)	—	—
Switzerland	1	1.11 (0.51–2.10)	—	—
Poland	1	2 (0.24–7.04)	1	2 (0.24–7.04)
Italy	1	0.64 (0.32–1.14)	1	0.99 (0.58–1.58)
Jordan	1	4.44 (1.94–8.57)	1	4.44 (1.94–8.57)
Iran	4	1.83 (0.75–3.35)	1	2.64 (1.37–4.56)
Crete	1	5.53 (4.34–6.92)	—	—
Saudi Arabia	—	—	1	0.00 (0.00–2.24)
Palestine	2	2.65 (1.68–3.83)	—	—
Tanzania	1	0.00 (0.00–3.52)	—	—
Nigeria	3	9.29 (1.94–3.08)	1	5.62 (3.74–8.08)
Madagascar	1	16.56 (11.21–23.18)	1	16.56 (11.21–23.18)
Japan	1	2.92 (1.88–4.32)	—	—
Australia	1	0.00 (0.00–0.47)	—	—
China	5	2.41 (0.61–5.35)	3	4.37 (0.54–11.54)

**Table 2:** Characteristics of the included studies in the meta-analysis based on eligibility criteria.

Study	Publication_year	Start_year	End_year	Type of study	Country	Mean age	Min_age	Max_age	Diagnosis_method	Total_sample_size	Total_PCR_positive	Total_culture_positive	Quality score
Calderaro et al. [16]	2018	2016	2018	Cross-sectional	Italy	3.6	0	14	Culture + PCR	1716	17	11	9
Hawash et al. [17]	2017	2016	2017	Cross-sectional	Saudi Arabia	29.9	0	60	PCR	163	0	—	10
Wang et al. [18]	2015	2010	2014	Cross-sectional	China	—	0	65	Culture	3224	—	9	9
Fiedoruk et al. [19]	2015	2010	2011	Cross-sectional	Poland		0	4	Culture + PCR	100	2	2	9
Assis et al. [20]	2014	2010	2011	Cross-sectional	Brazil	—	—	—	Culture + PCR	400	0	0	9
Hilmarsdóttir et al. [21]	2012	2003	2007	Cross-sectional	Iceland	—	0	83	Culture	464	—	3	10
El Qouqa et al. [22]	2011	2006	2007	Case-control	Palestine	5.01	0	12	Culture	600	—	16	9
Wang et al. [23]	2010	2004	2008	Cross-sectional	China	—	—	—	Culture	1152	—	14	8
Zheng et al. [9]	2008	2005	2008	Cross-sectional	China	—	0	83	Culture + real time PCR	2600	178	160	9
Maltezou et al. [24]	2001	1999	1999	Cross-sectional	Greece	—	0	14	Culture	132	—	2	8
Huhulescu et al. [25]	2007	2007	2009	Cross-sectional	Austria	37	0	89	Culture	306	—	1	10
Okwori et al. [1]	2009	2002	2004	Cross-sectional	Nigeria	—	18	—	Culture + PCR	480	27	27	9
Bucher et al. [4]	2008	2002	2002	Cross-sectional	Germany	—	—	—	Culture + real time PCR	22835	46	46	6
Bublitz et al. [26]	2014	2011	2011	Cross-sectional	Madagascar		0	15	Culture + PCR	163	27	27	10
Duan et al. [3]	2017	2010	2015	Cross-sectional	China	—	0	59	Culture + PCR	9208	80	80	10
Ehara et al. [27]	2000	1997	1999	Cross-sectional	Japan	3.56	0	14	Culture	821	—	24	7
Ternhag et al. [28]	2008	1997	2004	Cross-sectional	Sweden	36.5	0	100	Culture	101855	—	5133	7
Gijsbers et al. [29]	2011	2002	2004	Cross-sectional	Netherlands	8.8	4	16	Culture	220	—	1	6
Rees et al. [30]	2004	1998	1999	Cross-sectional	USA	—	—	72	Culture	1454	—	26	6
Vernacchio et al. [31]	2006	2001	2002	Case-control	USA	—	0	3	Culture + PCR	443	1	1	10
Talan et al. [32]	2001	1997	1998	Prospective case series	USA	2.9	4	41	Culture	549	—	4	9
de Wit et al. [33]	2001	1996	1999	Case-control	Netherlands	—	0	80	Culture	857	—	6	10
Karsten et al. [34]	2009	2004	2004	Case-control	Germany	—	0	80	Culture	1,046	—	10	9
Svenungsson et al. [35]	2000	1996	1997	Case-control	Sweden	41	15	98	Culture	851	—	2	9
Olesen et al. [36]	2005	2000	2001	Case-control	Denmark	1.2	0	4	Culture	424	—	10	9
Sinclair et al. [37]	2005	1997	1999	Cross-sectional	Australia	—	0	59	Culture	791	—	0	9
Jansen et al. [38]	2008	2005	2007	Prospective cohort	Germany	48	18	91	Culture/serology	104	—	6	9
Okwori. et al. [39]	2007	2005	2006	Cross-sectional	Nigeria	—	1	69	Culture	150	—	6	9
Omoigberale and Abiodun [40]	2002	2001	2001	Cross-sectional	Nigeria	—	1	59	Culture	215	—	47	9
Abdel-Haq et al. [41]	2006	1990	2002	Cross-sectional	USA	10.3	0	14	Culture	1920	—	201	10
Perdikogianni et al. [42]	2006	1993	2004	Cross-sectional	Crete	—	0	14	Culture	1285	—	71	5
Zheng et al. [43]	2007	2005	2006	Cross-sectional	China	—	2	83	Culture + real time PCR	700	52	52	10
Gasco et al. [44]	2000	1997	1997	Case-control	Tanzania	—	0	5	Culture	103	—	0	9
Stephen et al. [45]	2013	2011	2011	Cross-sectional	Switzerland	—	20	60	Culture	811	—	9	0
Garveriani et al. [46]	2007	2005	2006	Cross-sectional	Iran	—	0	5	Culture	405	—	13	9
GhasemiKebria et al. [47]	2010	2005	2006	Cross-sectional	Iran	5.07	0	22	PCR	455	12	—	9
Al Jarousha et al. [48]	2011	2006	2007	Case-control	Palestine	5.01	0	12	Culture	300	—	8	9
Huovinen et al. [49]	2010	2006	2006	Case-control	Finland	46.66	0	99	Culture	41841	—	406	8
Sihvonen et al. [50]	2009	2006	2006	Cross-sectional	Finland	—	—	—	Culture	41848	—	473	7
Orlandi et al. [51]	2001	1998	1999	Case-control	Brazil	0.86	0	5	Culture	130	—	1	10
Abdel-Haq et al. [52]	2000	1990	1997	Retrospective	USA	0.75	0	12	Culture	10 570	—	142	9
Nimri and Meqdam [53]	2004	2000	2002	Case-control	Jordan	48	12	84	Culture + PCR	180	8	8	10
Maraki et al. [54]	2003	1995	1999	Cross-sectional	Greece	—	0	59	Culture	7090	—	46	10
Torres et al. [55]	2001	1990	1994	Case-control	Uruguay	0.4	0	2	Culture	224	—	1	8
Dallal et al. [56]	2006	1998	1999	Cross-sectional	Iran	—	0	5	Culture	1600	—	11	10
Soltan Dallal et al. [57]	2004	2002	2002	Cross-sectional	Iran	3.24	0	12	Culture	300	—	8	10
Soleymani-Rahbar et al. [58]	2007	—	—	Cross-sectional	Iran	—	0	10	Culture	800	—	14	9

## Data Availability

The data are available from the corresponding author on reasonable request.

## Abbreviations

*Y. enterocolitica*: *Yersinia enterocolitica*
PRISMA: Preferred Reporting Items for Systematic Reviews and Meta-Analyses
TZ: Tayebeh Zeinali
SMR: Seyed Mohamad Riahi
EA: Ehsan Ahmadi
HDI: Human development index
WHO: World Health Organization
SD: Standard deviation
PCR: Polymerase chain reaction
Fig: Figure
USA: United States of America.

## Supplementary Materials

Supplementary Materials The search strategy is presented in the supplementary material.
