# Supplementary material for: Global Prevalence of Yersinia enterocolitica in Cases of Gastroenteritis: A Systematic Review and Meta-Analysis
Source: Int J Microbiol. 2021 Sep 2;2021:1499869. doi: 10.1155/2021/1499869 (PMC8433020; doi:10.1155/2021/1499869)
Supplement: Supplementary Materials — The search strategy is presented in the supplementary material. [file 1499869.f1.docx]

Supplementary file: search strategy

| Search strings Hits | | |
| --- | --- | --- |
| PubMed | | |
| #1 | Search yersinia enterocolitica [MeSH Terms] | 6245 |
| #2 | Search yersinia pseudotuberculosis [MeSH Terms] | 2935 |
| #3 | Search Gastroenteritis [MeSH Terms] | 229391 |
| #4 | Search Gastroenteritis[Title/Abstract] | 20708 |
| #5 | Search Yersiniosis [MeSH Terms] | 10818 |
| #6 | Search prevalence[MeSH Terms] | 2834532 |
| #7 | Search yersinia pestis [MeSH Terms] | 5065 |
| #8 | Search Plague [MeSH Terms] | 11516 |
| #9 | Search Humans [MeSH Terms] | 19531614 |
| #10 | Search (#1 OR #2) | 8450 |
| #11 | Search (#3 OR #5) | 239542 |
| #12 | Search (#10 AND #11) | 243548 |
| #13 | Search (#12 AND #6) | 946 |
| #14 | Search (#13 NOT #7 NOT #8) | 930 |
| #15 | Search (#14 AND #9) | 762 |
| #16 | Limit #15 to : year=2000-2019 | 315 |
| #17 | Limit #16 to Humans | 263 |

| Search strings Hits | | |
| --- | --- | --- |
| Embase | | |
| #1 | Search yersinia enterocolitica | 7858 |
| #2 | Search yersinia pseudotuberculosis | 3318 |
| #3 | Search yersinia infections | 2270 |
| #4 | Search yersiniosis | 2556 |
| #5 | Search gastroenteritis | 38757 |
| #6 | Search prevalence | 867139 |
| #7 | Search yersinia pestis | 6168 |
| #8 | Search plague | 12640 |
| #9 | Search pig | 393202 |
| #10 | Search #1 OR #2 OR #3 #4 OR #5 AND NOT #7 | 51258 |
| #11 | Search #10 AND #6 | 3032 |
| #12 | Search #11 AND NOT #8 | 2866 |
| #13 | Search #12 AND NOT #9 | 2690 |
| #14 | Search #13 AND NOT animals | 263 |
| #15 | Search #14 AND Humans | 30 |
| #22 | Search #15 AND year=2000-2019 | 20 |

| Search strings Hits | | |
| --- | --- | --- |
| Science direct | | |
| #1 | Search yersinia enterocolitica | 11149 |
| #2 | Search yersinia pseudotuberculosis | 4112 |
| #3 | Search yersinia infections | 19180 |
| #4 | Search yersiniosis | 1541 |
| #5 | Search gastroenteritis | 42938 |
| #6 | Search [Title, abstract or author-specified keywords] prevalence | 167311 |
| #7 | Search yersinia pestis | 6837 |
| #8 | Search [All fields] plague | 90040 |
| #9 | Search [Title] pig | 551076 |
| #10 | Search #1 OR #2 OR #3 #4 OR #5 | 53333 |
| #11 | Search #10 AND #6 | 2087 |
| #12 | Search #11 AND NOT #8 | 2086 |
| #13 | Search #12 AND NOT #9 | 2026 |
| #14 | Search #13 AND NOT [in Title, abstract or author-specified keywords] animals | 1752 |
| #15 | Search #14 AND[in Title, abstract or author-specified keywords] Humans | 371 |
| #16 | Search #15 AND year=2000-2019 | 319 |
| #22 | Search #15 AND NOT #7 | 318 |

| Search strings Hits | | |
| --- | --- | --- |
| Scopus | | |
| #1 | Search TITLE-ABS-KEY (yersinia enterocolitica) | 30119 |
| #2 | Search TITLE-ABS-KEY (yersinia) | 68520 |
| #3 | Search TITLE-ABS-KEY (infections) | 4554679 |
| #4 | Search TITLE-ABS-KEY (yersinia pseudotuberculosis) | 13211 |
| #5 | Search TITLE-ABS-KEY (yersinia infections) | 49762 |
| #6 | Search TITLE-ABS-KEY (yersiniosis) | 3710 |
| #7 | Search TITLE-ABS-KEY (gastroenteritis) | 39267 |
| #8 | Search TITLE-ABS-KEY (prevalence) | 979755 |
| #9 | Search TITLE-ABS-KEY (yersinia pestis) | 22160 |
| #10 | Search TITLE-ABS-KEY (plague) | 68461 |
| #11 | Search TITLE-ABS-KEY (pig) | 1210666 |
| #12 | Search TITLE-ABS-KEY (#1 OR #4 OR #5 OR #7) | 117466 |
| #13 | Search TITLE-ABS-KEY (#12 OR #6) | 119645 |
| #14 | Search TITLE-ABS-KEY ( #13 AND #8) | 9042 |
| #15 | Search TITLE-ABS-KEY ( #14 AND NOT #9 AND NOT #10 AND NOT #11) | 8433 |
| #16 | search #15 AND Humans | 5250 |
| #17 | Search #16 AND NOT animals | 4834 |
| #18 | Search #17 AND DOCTYPE ( article OR review ) AND PUBYEAR > 1999 AND PUBYEAR < 2020 | 4434 |
|  |  |  |
|  |  |  |
